# DQB1*06:02-Associated Pathogenic Anti-Myelin Autoimmunity in Multiple Sclerosis-Like Disease: Potential Function of DQB1*06:02 as a Disease-Predisposing Allele

**DOI:** 10.3389/fonc.2014.00280

**Published:** 2014-10-16

**Authors:** Nathali Kaushansky, Avraham Ben-Nun

**Affiliations:** ^1^Department of Immunology, The Weizmann Institute of Science, Rehovot, Israel

**Keywords:** MS/EAE, antigens/peptides/epitopes, neuroimmunology, T-cells, HLA-DR15-Tg mice

## Abstract

Susceptibility to multiple sclerosis (MS) has been linked mainly to the HLA-DRB1 locus, with the HLA-DR15 haplotype (DRB1*1501-DQA1*0102-DQB1*0602-DRB5*0101) dominating MS risk in Caucasians. Although genes in the HLA-II region, particularly DRB1*1501, DQA1*0102-DQB1*0602, are in tight linkage disequilibrium, genome-wide-association, and gene candidate studies identified the DRB1*15:01 allele as the primary risk factor in MS. Many genetic and immune-functional studies have indicated DRB1*15:01 as a primary risk factor in MS, while only some functional studies suggested a disease-modifying role for the DRB5*01 or DQB1*06 alleles. In this respect, the susceptibility of DRB1*15:01-transgenic (Tg) mice to myelin basic protein- or myelin oligodendrocyte glycoprotein-induced MS-like disease is consistent with primary contribution of DRB1*15:01 to HLA-DR15+ MS. The studies summarized here show that susceptibility to MS-like disease, induced in HLA-“humanized” mice by myelin oligodendrocytic basic protein or by the proteolipid protein, one of the most prominent encephalitogenic target antigens implicated in human MS, is determined by DQB1*06:02, rather than by the DRB1*15:01 allele. These findings not only offer a rationale for a potential role for DQB1*06:02 in predisposing susceptibility to MS, but also suggest a more complex and differential functional role for HLA-DR15 alleles, depending on the primary target myelin antigen. However, the conflict between these findings in HLA-Tg mice and the extensive genome-wide-association studies, which could not detect any significant effect from the DQB1*06:02 allele on MS risk, is rather puzzling. Functional analysis of MS PBLs for DQB1*06:02-associated anti-myelin autoimmunity may indicate whether or not DQB1*06:02 is associated with MS pathogenesis.

## Introduction

Multiple sclerosis (MS), the most common neurological disease affecting young adults in the Western world, is a CNS demyelinating disease associated with complex pathogenic autoimmunity against several CNS-myelin target antigens (1). Although the etiology of MS is yet unknown, numerous studies have confirmed a strong genetic component underlying its etiology. Susceptibility to MS has been associated with genes within the major histocompatibility complex have been recognized for several decades, particularly with the genes of the HLA-DR2 serotype (DR15 haplotype) (2). The HLA-DR15 association with MS susceptibility was refined later on, using DNA-based typing methods, to the HLA-DRB1*1501 locus (3). More recent genome-wide-association studies (GWASs), which identified several MHC genes and over 100 non-MHC genetic variants/genes associated with MS susceptibility (4) have localized the greatest effect on MS risk to the HLA-DRB1 locus, with the DRB1*15:01 allele within the HLA-DR15 haplotype bearing the strongest association with MS. However, the HLA-DR15 haplotype genes (DRB1*15:01, DQA1*01:02, DQB1*06:02, and DRB5*01:01) are in strong linkage disequilibrium (LD). The remarkable LD between these genes makes it difficult to determine by genetic studies whether the functionally relevant effect on MS derives from DRB1*15:01 only, or also from its neighboring DQA1*01:02-DQB1*06:02 or DRB5*01:01 genes of HLA-DR15 haplotype.

The availability, of “humanized” mice [HLA-transgenic (Tg) mice] that were genetically manipulated to express either of the human DRB1*15:01, DQA1*01:02-DQB1*06:02, or DRB5*01:01 molecules, provide a powerful tool to discern the potential contribution of each of these three sets of genes, individually or in combination (epistasis), to the development of MS.

Most previous genetic and immunological studies suggested DRB1*15:01 as the disease-predisposing gene in MS (5, 6) are in agreement with the GWASs, suggesting that greatest risk to MS is driven by the HLA-DRB1*15:01 allele, while association to MS of other alleles of DR15 haplotype is only secondary to their LD with HLA-DRB1*15:01 allele (4, 7). The HLA-II-Tg mice studies showing that HLA-DRB1*1501-Tg mice are susceptible to myelin basic protein (MBP)- or myelin oligodendrocyte glycoprotein (MOG)-induced experimental autoimmune encephalomyelitis (EAE) (8, 9) are also consistent with the primary contribution of DRB1*1501 to MS pathogenesis. However, we recently showed that “humanized” mice expressing DQB1*06:02, but not the DRB1*15:01, genes can develop MS-like disease mediated by autoimmune cells reactive against a CNS protein, myelin oligodendrocytic basic protein (MOBP) (10), which is also an important target antigen for the immune attack in MS. These findings were the first to implicate DQB1*06:02-associated immune cells in the pathogenesis of MS, and to suggest that DQB1*06:02 is also a potential disease-predisposing gene. The DQB1*06:02-associated pathogenic autoimmunity against MOBP target antigen appeared not to be a limited case. We found that the pathogenic autoimmunity against the proteolipid protein (PLP), a highly encephalitogenic target antigen in MS, is also determined by HLA-DQB1*06:02, and not by HLA-DRB1*15:01 allele (10, 11).

Here, we review our and others’ studies, which implicate pathogenic DQB1*06:02-associated autoimmunity in the pathogenesis of MS and highlight the possibility that DQB1*06:02 is also an important disease-predisposing allele, rather than just disease-modifying as previously perceived (10–14).

## HLA-DR15 Haplotype and MS

Multiple sclerosis is associated with complex pathogenic autoimmunity directed against CNS components, with several of the myelin proteins, such as MBP, PLP, MOG, and MOBP, being the major potential target antigens in MS. The genetics of MS is complex; in recent GWASs, several HLA and over 100 non-HLA genes have been associated with the disease, with the DRB1*15:01 within the extended HLA(-DQB1*06:02, -DQA1*01:02, -DRB1*15:01, -DRB5* 01:01) haplotype (“HLA-DR15” haplotype thereafter) bearing the strongest association to MS (4, 15, 16). Nevertheless, extensive LD among these loci, makes it difficult not unequivocally establish by fine-genetic mapping studies whether the functionally relevant effect on MS derives from DRB1*15:01, DQA1*01:02, DQB1*06:02, or DRB5*01:01 loci of HLA-DR15, their combination, or from their epistatic interactions. Although many previous genetic studies indicated DRB1*15:01 as the primary risk factor in MS among the Caucasian populations (5, 17, 18), studies with unique populations show selective association with DRB1*15:01 independent of DQB1*06:02 [African–Americans (19)]. In African–Brazilian MS patients, the association was with HLA-DQB1*06:02 rather than HLA-DRB1*15:01 (13). More recent studies showed that individuals with incomplete haplotypes bearing only HLA-DRB1*15:01 or HLA-DQB1*06:02 were not predisposed to MS; the HLA-DQA1*01:02, which by itself shows no primary association with MS, increases the risk of MS when combined with HLA-DRB1*15:01 in *trans*, and plays a protective role in the absence of HLA-DRB1*15:01 (20). This study also suggested that all three HLA-DRB1, HLA-DQA1, and HLA-DQB1 alleles can affect MS susceptibility through epistatic interactions (20). In addition to HLA-DRB1*15:01, there are other HLA-DRB1 alleles that can also increase MS risk [such as HLA-DRB1*17:03 allele in the Swedish and Canadian MS populations (21)], albeit to a lesser extent, while other HLA-DRB1 alleles, particularly HLA-DRB1*14 can abrogate the risk associated with HLA-DRB1*15 when they are inherited together (22), apparently via functional epistasis. Thus, the effect of the MHC on susceptibility to MS appears quite complex as it may involve also complex interactions between alleles of HLA haplotypes through *cis* or *trans* functional epistatic interactions, suggesting the significance of haplotypic rather than allelic association between HLA and MS. Yet, the HLA-DR15 haplotype, comprising the DRB1*15:01, DQA1*01:02, DQB1*06:02, and DRB5*01:01 alleles, is the most prevalent haplotype (~30%) among MS patients of the Western population.

## HLA-DQB1*06:02 Allele in Unique Populations with MS

While most MS genetics studies in North European, Caucasian populations suggest the HLA-DRB1*15:01 as the genetic risk factor in MS (5), Spurkland, Thorsby, and Vartdal studied the specific HLA-DR and DQ alleles of 181 Norwegian MS patients. Patients who carried DQB1*06:02 or 06:03 without DRB1*15:01 were identified, but none who were DRB1*15:01 without DQB1*06:02 (23) were identified. Related observations were made in a relatively small cohort of Hong Kong Chinese patients with MS. In this specific Chinese patients, DR15 is expressed without DQB1*06:02 and DQB1*06:02 without DR15, so that one can ask whether either or both of the dissociated genes appear to confer an enhanced risk. It was found that the enhanced risk to MS was associated with DQB1*06:02 (12). A similar case has been made for MS susceptibility in Afro-Brazilians, where the frequency of DQB1*06:02 among patients is higher than for the main DR15 allele in that unique population, DRB1*15:03 (13). Hence, in African populations that show greater haplotypic diversity than European and also distinct patterns of LD, it was possible to separately appraise the relative contributions of HLA-DR and DQ sequences to susceptibility. A caveat here is the analysis of HLA-DR and DQ associations conducted in a large cohort of African American MS patients, where a selective association with HLA-DRB1*15:01 and not with HLA-DQB1*06:02 was identified (19). Another study in MS patients from Malaga, Spain, that showed association of HLA-DQB1*06:02 with MS in the absence of DRB1*15:01 (14) is in line with the findings in the African Brazilians. These studies suggest that HLA DRB1*15:01 may not be the sole determinant of MS susceptibility.

## Myelin T-Cell Epitopes in HLA-Tg Mice

Immunodominant epitopes in MS are also often encephalitogenic epitopes in relevant susceptible experimental animals [e.g., MBP 89-101 in SJL/J mice (24), PLP 43-64 in Pl/J mice (25), MOG 35-55 in H-2^b^ mice (26, 27), Lewis rats (28) and Rhesus monkeys (29), and MOBP 65-86 in H-2^b^ mice (30)]. Nevertheless, these animal models do not fully reflect the immune responses seen in MS. In this regard, HLA-Tg mice are a more powerful tool for the analysis of human T-cell responses to myelin epitopes, their association with particular HLA-restriction elements, and their pathogenic credentials. Proof of principle that human T-cell responses can be studied in the “humanized” HLA-Tg mouse model was provided by the demonstration of T-cell responses to MBP 139-154 in HLA-DR Tg mice (8) and the induction of EAE with MBP 84-102 in HLA-DR2-transgenic mice, which also express a human TCR recognizing the MBP epitope (31).

In the study of Sireci et al., MBP13–33, 139–154, and 87–106, reported as being presented by different HLA class II alleles, were tested for their ability to induce HLA restricted proliferative responses in HLA-DR1 Tg mice (32). Of these, only MBP13–33 induced severe EAE in the HLA-DR1 Tg mice (32); MBP87–106, which is an immunodominant MBP epitope in MS, induced only weak responses and very mild EAE (32). A more comprehensive study in HLA-Tg of the HLA-restriction of MBP epitopes was conducted by Das et al. (33). In their study, the reactivity to overlapping peptides spanning the MBP molecule by cells isolated from myelin-primed mice singly transgenic for HLA-DR3, -DQ6, or -DQ8, or double transgenic for HLA-DR3/DQ6 or -DR3/DQ8, was investigated, and a strong T-cell response to MBP81-100 was observed in HLA-DR3/DQ8 Tg mice resulting in severe EAE (33).

Reports on the association of PLP epitopes with specific HLA alleles in HLA-Tg mice are scarce. PLP175-192 considered as immunodominant in HLA-DR4 individuals could induce a strong response accompanied with clinical symptoms of EAE in HLA-DR4-IE Tg mice (34); T-cell lines to the HLA-DR2 MS-associated PLP95-116 that were raised from HLA-DR2 Tg mice could induce neurological impairment upon transfer into these mice (10, 35). More recently, TCR^PLP45–53^ Tg mice were generated and were used to prepare the HLA-A3/TCR^PLP45–53^ double Tg mice (36). The most comprehensive studies in HLA-Tg mice have been conducted to identify the immunodominant epitopes of MOG (37, 38). In the study of Khare et al. (38), HLA-Tg (-DR2, -DR3, -DR4, -DQ6-601, -DQ6-604, and -DQ8) mice were immunized with recombinant MOG (rMOG) and the primed lymph node cells tested for reactivity to overlapping peptides. All six transgenics showed strong responses to MOG1–20, 31–50, and 61–80, which were shown as immunodominant epitopes in MS (39). HLA-DR2 Tg mice also reacted to MOG 91-110, which is immunodominant in the T-cell response to MOG in rhesus monkeys with MOG-induced EAE (29); HLA-DR4 Tg mice reacted to MOG31–50 and 91–110 and the potential relevance of this latter epitope to MS pathogenesis was confirmed by the study of Forsthuber et al. (37). They showed that HLA-DR4 Tg mice immunized with rMOG respond predominantly to MOG 97–108 and develop severe EAE (37). Another example of clinical EAE in HLA-Tg mice is the study reporting MOG35–55-induced EAE in HLA-DR2 Tg mice (9).

Various examples of TCR transgenics expressing MS patient-derived TCRs (31, 36, 40), or expressing murine TCR-Tg of pathogenic T-cells (41, 42) have been described. Several new observations were made in humanized TCR models, progressing translational, and understanding of MS T-cell responses beyond the generic observations that have been made with standard murine EAE models. The humanized transgenic models provide information that could not be obtained using assays of patient-derived cells, e.g., pathogenic credentials of autoimmune response to myelin epitopes. For many years, studies have defined patterns of autoimmune anti-myelin T-cell responses of MS patients. Some myelin epitopes were identified to be more disease specific than others, and some were more clearly related to disease progression than others. However, as the responses in MS are assessed in *ex vivo* cultures, it has not been possible to attribute functional, pathogenic outcomes to these responses. For this purpose, HLA-Tg mice genetically manipulated to express TCRs specific to myelin epitope(s) that develop spontaneous MS-like disease are instrumental.

Strikingly, none of the reported humanized TCR transgenics express TCR specific for an HLA-DQ6-restricted myelin epitopes. Furthermore, and quite surprisingly, extensively reviewing the literature on studies devoted to defining MS-relevant myelin epitopes in humans (MS) revealed that little importance was given to DQ6-presented epitopes, as the vast majority of studies reported only DR15-associated epitopes. A similar situation was noticed in the studies on humanized HLA-Tg mice. Only one group (38, 43) studied EAE in HLA-DQ6-Tg mice, and analyzed epistatic effects between DQ6 and DR alleles on PLP-induced EAE, although none of the DQ and DR alleles in these studies (32, 33, 43, 44) were of the HLA-DR15 haplotype, the most prevalent haplotype in MS. Thus, it appears that the HLA-DQ6-associated autoimmune reactivity against CNS antigens has only been poorly explored, particularly the autoimmunity associated with DQB1*06:02 molecule of the HLA-DR15 haplotype. The reasons are not clear, and are even less understood, as DQ6-associated anti-myelin autoreactivity may also play a role in the pathogenesis of MS, as suggested from studies in some unique populations (12–14) or from our study showing that DQB1*06:02, but not DRB1*15:01, determine disease susceptibility to MOBP or PLP in HLA-Tg mice (10, 11).

## HLA-DQB1*06:02-Associated Susceptibility to MS-Like Disease Induced by Human MOBP in HLA-Transgenics

MOBP is a CNS-myelin-specific protein that plays a role in stabilizing the myelin sheath (45). Northern blot analysis, *in situ* hybridization, immunochemistry, and immunoelectron microscopy (45, 46) indicated that MOBP is expressed exclusively in CNS white matter, and more particularly throughout compact myelin, predominantly at the major dense lines of myelin (45). The encephalitogenic potential of MOBP was demonstrated in H-2^b^ mice with the MOBP65-88 being the encephalitogenic epitope (30), and in the H-2^s^ (47) mice with MOBP15-36 epitope being the immunodominant and encephalitogenic epitope. The relevance of the autoimmunity against these encephalitogenic epitopes to the pathogenesis of MS was investigated by analyzing the autoreactivity to overlapping human MOBP peptides by MS patients and healthy control individuals. In two separate studies (30, 47), analysis of the proliferative response to MOBP peptides by PBLs from MS patients and controls revealed autoreactivity to MOBP1–23, MOBP15–36, and MOBP65–87 epitopes in MS patients and controls. Although a significant difference between MS patients and controls could not be determined, two of the epitopes that were detected by the PBLs, MOBP15–36 and MOBP65–87, were found encephalitogenic for mice, suggesting the relevance of autoimmunity against MOBP15–36 and MOBP65–87 epitopes to MS pathogenesis (30, 47). In another study, Holz et al. (48) showed proliferative response to several human MOBP epitopes by PBLs from relapsing/remitting MS patients and controls, with reactivity to MOBP21-39 somewhat higher in MS patients (48).

We used HLA-Tg mice expressing either the DRB1*15:01 or DQB1*06:02 heterodimers, as well as (DRB1*15:01 × DQB1*06: 02)F1 double Tg mice (10), in order to illuminate the relative potential contribution of each of these molecules, or their co-expression, to MOBP-associated pathogenic autoimmunity in MS patients with the “HLA-DR15 haplotype.” We showed (10) that while the HLA-DRB1*15:01 transgenics are refractory to disease induction, the HLA-DQB1*06:02 Tg mice are susceptible to EAE induction by hMOBP, through pathogenic T-cells reactive against MOBP15–36 and MOBP55–77 encephalitogenic epitopes. HLA(DRB1*15:01 × DQB1*06:02)F1 double Tg mice were also susceptible to EAE induction by hMOBP, although the DRB1*15:01/MOBP15–36 reactive T-cells are anti-inflammatory. The presence of the HLA-DRB1*15:01 gene product in the HLA-(DRB1*15:01 × DQB1*06:02)F1 double Tg mice did not impose any immunomodulatory effect. The pathology was essentially similar between HLA-DQB1*06:02 and HLA (DRB1*15:01 × DQB1*06:02) F1 double Tg mice, and pathological manifestations were highly reminiscent of MS. Both immunogenic peptides (MOBP15–36 and MOBP55–77) caused overt clinical EAE associated with perivascular and parenchymal infiltrates, widespread demyelination, axonal loss, and optic neuritis with a high degree of demyelination in the optic nerve (10). This study, which presented a new “humanized” model of MS-like disease provided the first evidence of pathogenic HLA-DQ-associated anti-myelin autoimmunity, and further implicated the autoimmunity against MOBP in the pathogenesis of MS (10).

## HLA-DQB1*06:02 Determines Susceptibility to MS-Like Disease Induced by PLP in HLA-DR15 Transgenic Mice

While our recent study (10) showing that MOBP can induce MS-like disease in HLA-DQB1*06:02-Tg mice offers a rationale for the HLA-DQB1*06:02 association with MS, it raised the question of whether DQ6-autoimmunity against other myelin/neuronal target antigens/epitopes can be pathogenic and may also play a role in pathogenesis of MS. PLP autoimmunity has been strongly implicated in MS. Some functional studies have also attempted to correlate immune reactivity with MS relapse (49). We therefore investigated the pathogenic potential of PLP in HLA-Tg mice expressing the DRB1*15:01 and DQB1*06:02 gene products of the HLA-DR15 haplotype. Our results (11) showed that the DQB1*06:02-associated pathogenic autoimmunity against MOBP is not a limited case, and that DQB1*06:02-autoimmunity against other CNS antigens may also play a role in the pathogenesis of MS. We showed that susceptibility to a “humanized” MS-like disease induced by highly encephalitogenic protein, PLP, which is the most abundant CNS protein and one of the most prominent target antigens implicated in human MS, is determined by HLA-DQB1*06:02, and not by DRB1*15:01. Thus, the HLA-DRB1*15:01 transgenics were found to be refractory to PLP disease induction, whereas the HLA-DQB1*06:02 transgenics were susceptible via T-cells reactive against PLP139-151 and PLP175-194 encephalitogenic epitopes. Although both transgenics react against these epitopes, the PLP139–151 and PLP175–194-reactive T-cells are of Th2-type in HLA-DRB1*15:01 transgenics, and pathogenic Th1/Th17-type cells in the HLA-DQB1*06:02 transgenic mice. The PLP-induced EAE in HLA-DQB1*06:02 transgenic mice showed a typical caudo-rostral clinical progression that was associated with CNS demyelination, axonal damage, and with optic neuritis. However, unlike usually observed in PLP-induced disease or other EAE models in the wild type mice, the CNS pathology in phPLP175–194-induced EAE in HLA-DQB1*06:02 transgenics was more pronounced in the brain rather than in the spinal cord (11). Such a strong involvement of cerebellum and brainstem exceeding that of spinal cord, which has been suggested by Stromnes et al. (50) to be a feature of Th17-driven disease, corresponded to the high Th17 secretion by PLP175–194-primed LNC derived from DQB1*06:02-Tg mice.

## Summary

HLA-DR15-Tg mice are instrumental in investigating the encephalitogenic potential of myelin/neuronal epitopes relevant to HLA-DR15+ MS in the context of the genes encoding the individual molecules comprising the HLA-DR15 haplotype. While the previously reported susceptibility of DRB1*15:01-Tg mice to MBP- or MOG-induced EAE is consistent with the primary contribution of DRB1*15:01 to MS pathogenesis, our recent studies in HLA-Tg mice showed that DQB1*06:02 but not DRB1*15:01 determines pathogenic autoimmunity against PLP as well as against MOBP (10, 11). These findings provide a rationale, and mechanisms, for the involvement of HLA-DQB1*06:02-associated pathogenic autoimmunity in the pathogenesis of MS, thereby, implicating the HLA-DQB1*06:02 in the genetic susceptibility to MS. This places MS more firmly in the group of autoimmune diseases in which a functional association is presumed, including type I diabetes and celiac disease (40, 51). The findings showing that DQB1*06:02 determines pathogenic autoimmunity against PLP (11) and MOBP (10) together with previously reported studies showing that DRB1*15:01 determines pathogenic autoimmunity against MBP and MOG (8, 9) suggest a more complex and differential genetic predisposition to HLA-DR15+ MS, depending on the primary CNS target antigen/epitope against which the pathogenic autoimmunity is primarily directed (or triggered). It is quite puzzling, however, that the pathogenic DQB1*06:02-associated anti-myelin autoimmunity that emerged from functional studies in HLA-Tg mice is not reflected by the extensive GWASs in MS, which determined the DRB1*15:01 as the major risk allele in MS without any significant association of DQB1*06:02 with MS risk. Whether the pathogenic DQB1*06:02-associated autoimmunity is limited to the reductionist transgenic models of MS, or whether GWASs could not distinguish the DQA1*01:02- and DQB1*06:02-related functionally relevant effects on MS risk from the functional effects driven by DRB1*15:01 allele, due to strong LD, would require functional studies with PBLs of HLA-DR15^+^ MS patients, in particular PBLs reactive against MOBP and/or PLP.

## Take Home Messages

- The susceptibility to MS-like disease induced by MBP or MOG in HLA-Tg mice is determined by DRB1*15:01, while disease induced by MOBP or PLP is controlled by DQB1*06:02.
- These findings have important bearing on the candidacy of the DQB1*06:02 allele as genetic risk factor for MS.
- These findings in transgenic models also suggest a more complex and differential functional role for HLA-DR15 genes in determining susceptibility to MS, depending on the target brain-myelin antigen.

## Conflict of Interest Statement

The authors declare that the research was conducted in the absence of any commercial or financial relationships that could be construed as a potential conflict of interest.

## Abbreviations

EAE, experimental autoimmune encephalomyelitis; GWASs, genome-wide-association studies; LD, linkage disequilibrium; MBP, myelin basic protein; MOBP, myelin-associated oligodendrocytic basic protein; MOG, myelin oligodendrocyte glycoprotein; MS, multiple sclerosis; PLP, proteolipid protein; Tg, transgenic mice.
